# Characterization of pTS14, an IncF2:A1:B1 Plasmid Carrying *tet*(M) in a *Salmonella enterica* Isolate

**DOI:** 10.3389/fmicb.2020.01523

**Published:** 2020-07-03

**Authors:** Ying-ying Liu, Xiao-kang Liu, Xiao-die Cui, Min Chen, Shuai-hua Li, Dan-dan He, Jian-hua Liu, Li Yuan, Gong-zheng Hu, Yu-shan Pan

**Affiliations:** College of Animal Husbandry and Veterinary Science, Henan Agricultural University, Zhengzhou, China

**Keywords:** *Salmonella enterica*, *tet*(M), Tn*6709*, biological features, IncF2:A1:B1 plasmid

## Abstract

The objective of this study was to explore the genetic and biological features of the *tet*(M)-harboring plasmid pTS14 in *Salmonella enterica* strain S14 isolated from a chicken fecal sample. Plasmid pTS14 was identified by conjugation, S1-pulsed-field gel electrophoresis (PFGE), Southern hybridization, and plasmid sequencing. The biological characteristics of pTS14 were assessed via stability, growth kinetics, and starvation survival experiments. Strain S14, belonging to ST3007, harbored a 119-kb *tet*(M)-bearing IncF2:A1:B1 conjugative plasmid pTS14. The plasmid pTS14 contained a novel transposon Tn*6709* with the genetic structure IS*26*-*tnpA1*-*tnpA2*-Δ*orf13*-*LP*-*tet*(M)-*tnpX*-Δ*tnpR*-IS*26*, and the resistance genes *tet*(B), *tet*(D), *strAB*, *sul2*, and *bla*_TEM–1b_. In addition, pTS14 was found to be highly stable in the recipient strain *E. coli* J53. The transconjugant TS14 exhibited a higher survival ratio than *E. coli* J53 under permanent starvation-induced stress. The *tet*(M)-bearing IncF2 epidemic plasmid lineage may accelerate the dissemination of *tet*(M) and other genes by coselection, which could constitute a potentially serious threat to clinical treatment regimens.

## Introduction

The tetracycline resistance gene *tet*(M) encodes a ribosomal protection protein that confers tetracycline resistance to a variety of bacterial species (Franke and Clewell, 1981; Roberts et al., 1986; Donhofer et al., 2012; Roberts and Schwarz, 2016), including 38 genera of Gram-positive bacteria and 39 genera of Gram-negative bacteria, with most associated with other *tet* genes^1^, likely through the association with integrative and conjugative transposons located on the chromosome or conjugative plasmids, which facilitate horizontal transfer (Bryan et al., 2004; Jones et al., 2006; Tuckman et al., 2007; de Vries et al., 2009; Hu et al., 2013). In Gram-negative bacteria, the *tet*(M) gene was first reported in *Escherichia coli* in 2006 (Jones et al., 2006) and later described in *Salmonella enterica* isolates from chicken and pig feces in China in 2017 (Ma et al., 2017). To date, several plasmids harboring the *tet*(M) gene have been reported in *E. coli*, which include the IncHI2-type plasmids p1106 (MG825373), pECAZ147_2 (CP018993), and pTW4-IncHI2 (MK293945), as well as the hybrid IncN1-IncHI2-type plasmid pHN6DS (MH459020) and IncX1-FI:A:B plasmid pYPE12 (CP041443). The incompatibility group IncF is a main vehicle for the dissemination of the *rmtB* and/or *bla*_CTX–MS_ genes in *Enterobacteriaceae* (Deng et al., 2011; Hou et al., 2012). However, there are relatively few reports of *tet*(M)-harboring IncF plasmids from *Salmonella*. Here, we report the complete sequence of the *tet*(M)-harboring IncF2:A1:B1 plasmid pTS14 isolated from *S. enterica*. A novel transposon, Tn*6709* harboring the *tet*(M) gene, as well as three other resistance modules, were located on the same plasmid, pTS14. Moreover, the biological characteristics of plasmid pTS14 in *Salmonella* were further investigated.

## Materials and Methods

### Bacterial Strains

During a survey of the *tet*(M) gene in Henan Province, China, conducted in December 2017, one *tet*(M)-positive *Salmonella* strain, named S14, was isolated from the feces of a chicken. Strain identification was confirmed by PCR analysis and 16S rRNA sequencing along with MALDI-TOF MS detection (AXIMA Performance; Shimadzu Corporation, Kyoto, Japan) as described previously (Weisburg et al., 1991; Pavlovic et al., 2013). Subsequently, strain S14 was serotyped according to the Kauffmann–White scheme with the use of commercial antiserum.

### Susceptibility Testing and Detection of Tetracycline Resistance Genes

The susceptibility of *Salmonella* strain S14 to 13 antibiotics (Supplementary Table S1) was determined via the broth microdilution method and interpreted in accordance with the guidelines of the Clinical and Laboratory Standards Institute (2017). Minimum inhibitory concentrations were calculated on three independent occasions. *E. coli* ATCC 25922 was used as a quality control strain. The *tet*(A), *tet*(B), *tet*(C), and *tet*(D) genes were screened by PCR as described previously (Sun et al., 2018).

### Multilocus Sequence Typing (MLST)

To investigate the genetic typing of the isolate, MLST of seven housekeeping genes (*thr*A, *pur*E, *suc*A, *his*D, *aro*C, *hem*D, and *dna*N) was performed as described previously (Yap et al., 2016). The sequences were subsequently submitted to the MLST database^2^ and assigned existing or novel allele type identification numbers. The corresponding sequence types were derived from the set of allelic profiles of each of the seven loci.

### Conjugation Experiments

Mating experiments were conducted to evaluate the transferability of the *tet*(M) gene with the *tet*(M)-positive strain S14 as the donor and rifampicin-resistant *Salmonella* strain JS-500 and sodium azide-resistant *E. coli* strain J53 as the recipients. The transconjugants were selected on MacConkey agar plates supplemented with doxycycline (16 mg/L) and rifampicin (400 mg/L) for *Salmonella* JS-500 or doxycycline (16 mg/L) and sodium azide (200 mg/L) for *E. coli* J53. The conjugation frequency was calculated as the ratio of the number of transconjugants per recipient. All transconjugants were confirmed by pulsed-field gel electrophoresis (PFGE). The presence of the *tet*(M) and other *tet* genes in all transconjugants was confirmed by PCR analysis and sequencing.

### S1-PFGE and Southern Hybridization

Prior to PFGE, DNA from the donor strain S14 and the corresponding *E. coli* J53 transconjugant (named TS14) were treated with S1 nuclease. The location of *tet*(M) gene was identified by Southern blot hybridization with the use of a probe for the *tet*(M) gene.

### Plasmid Sequencing and Annotation

The plasmid from the transconjugant was extracted using the QIAGEN Plasmid Midi Kit (Qiagen, Hilden, Germany) and sequenced with Illumina Hiseq technology (Illumina, Inc., San Diego, CA, United States). Assembly of the generated sequences with Newbler software v2.6 (Roche Diagnostics Corporation, Indianapolis, IN, United States) generated nine contigs. Gaps between the contigs were closed by PCR and sequencing. The plasmid sequences were initially predicted and annotated using the Subsystem Technology (RAST v2.0) server (Aziz et al., 2008), and corrected manually using the BLASTn and BLASTp algorithms^3^. The plasmid replicon genotype was identified using the PlasmidFinder database^4^. Comparative analysis and generation of plasmid maps were performed using the Python application Easyfig and the BLAST Ring Image Generator (Alikhan et al., 2011; Sullivan et al., 2011).

### Biological Characteristics of the *tet*(M)-Harboring Plasmid pTS14

To assess bacterial growth kinetics, the transconjugant TS14 and recipient *E. coli* J53 were incubated overnight at 37°C in lysogeny broth (LB; 5 mL) and then diluted to an optical density at 600 nm (OD_600_) of 0.004 in 20 mL of fresh LB with or without doxycycline (16 mg/L). Over a 15-h inoculation period, bacterial growth was measured and recorded every hour. To evaluate plasmid stability, transconjugant *E. coli* TS14 was maintained for 14 days in daily refreshed LB (100-fold dilution) without antibiotic selection, as previously described (Wu et al., 2018). Approximately 100 colonies were randomly chosen and replica plated onto LB agar plates with doxycycline. All colonies grown on doxycycline-supplemented agar were subjected to PCR analysis to confirm the presence of the *tet*(M) gene. M9-glycerin (0.2%) minimum medium was used for starvation survival experiments of *E. coli* J53, TS14, and a mixture of both. The percent survival was calculated as the ratio of the mean number of CFUs divided by the number of CFUs after overnight incubation for each of the triplicate suspensions on days 1, 2, 3, 5, 7, 10, and 15. Experiments were repeated in three separate assays.

### Nucleotide Sequence Accession Number

The complete sequence of plasmid pTS14 was submitted to the GenBank under the accession number MN328348.

## Results and Discussion

### Characterization of *Salmonella* Strain S14

The *tet*(M)-positive strain S14 was serotyped as *S. enterica*, belonging to ST3007. The results of the conjugation assay indicated that the *tet*(M) gene can be successfully transferred to *Salmonella* JS-500 and *E. coli* J53 at frequencies of 1.638 × 10^–4^ and 1.397 × 10^–4^, respectively, which were assigned as TS14-JS500 and TS14. Susceptibility testing showed that S14, TS14-JS500, and TS14 were all resistant to doxycycline, tetracycline, gentamicin, sulfamethoxazole-trimethoprim, and amoxicillin (Supplementary Table S1). The transconjugant TS14 was selected for further study. S1-PFGE and Southern hybridization indicated that the *tet*(M) gene was located on a plasmid of ∼119 kb in size, designated as pTS14 (Supplementary Figure S1). The results of the mating experiments demonstrated that plasmid pTS14 harboring *tet*(M) can be self-transmissible and spread between and across genera.

### Characterization of Plasmid pTS14

**FIGURE 1:**
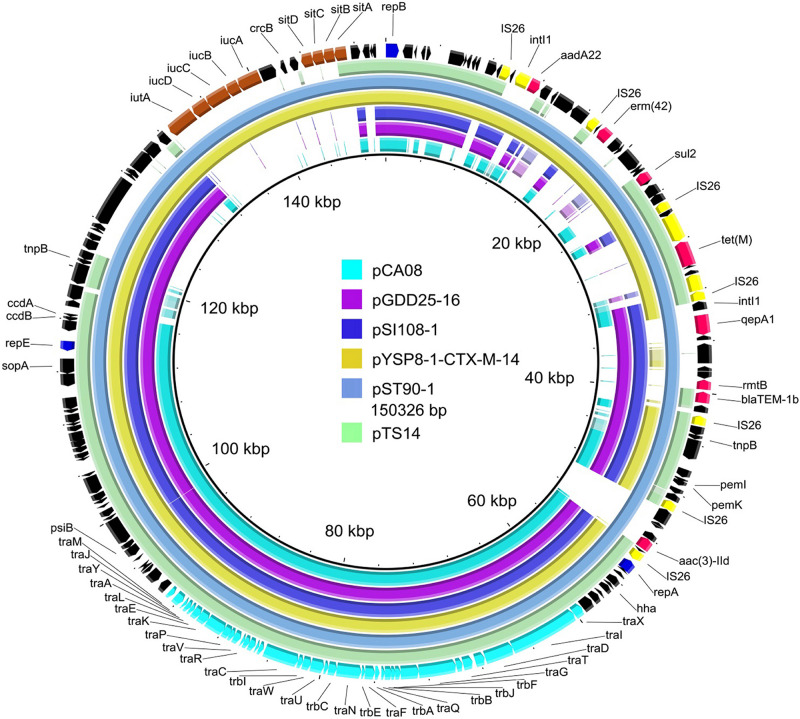
Whole-plasmid sequence of pTS14 and comparison of similar IncF2 plasmids. The 150326-bp pST90-1 was used as a reference plasmid at the highest coverage (79%). Key features of pST90-1 are highlighted in different colors. Replicon genes are in blue; transfer-associated genes in cyan; resistance genes in red; mobile elements in yellow; the loci of stability-associated genes (*iut*A-*iuc*ABCD and *sit*ABCD) in maroon; and hypothetical proteins in black. The outer ring comprises the CoDing sequence of pST90-1. The plasmids in this study included pTS14 (IncF2:A1:B1, MN328348), pST90-1 (IncF2:A1:B58, CP050735), pYSP8-1-CTX-M-14 (IncF2:A1:B1, CP037912), pSI108-1 (IncF2:A1:B58, CP050770), pGDD25-16 (IncF2:A1:B1, MH316135), and pCA08 (IncF2:A16:B20, CP009233).

Sequencing of the plasmid pTS14 from transconjugant TS14 revealed a 119,493-bp circular episome with a mean G + C content of 50.81% with 155 putative open reading frames (ORFs). The plasmid pTS14, belonging to IncF2:A1:B1, has a typical FII-type backbone and three conserved modules (Figure 1): (i) regions encoding the plasmid replication gene *rep*A; (ii) regions responsible for plasmid transfer and two conserved pilus-encoding loci (*tra* and *trb*); (iii) plasmid maintenance region, plasmid segregation system (including *Par*AB and *Psi*AB) and a multiple toxin-antitoxin (TA)-based addiction system (including *Pem*I/*Pem*K, *Ccd*A/*Ccd*B, and *Hok*); and two accessory regions (regions encoding antimicrobial resistance and virulence).

As shown in Figure 1, BLASTn comparisons demonstrated that plasmid pTS14 (IncF2:A1:B1) shared high homology to *S*. *enterica* serovar *Typhimurium* plasmid pST90-1 (IncF2:A1:B58, CP050735) with 99.97% identity at 81% coverage and to *E. coli* plasmid pYSP8-1-CTX-M-14 (IncF2:A1:B1, CP037912) with 100% identity at 80% coverage. In general, the incompatibility group IncFII is an epidemic plasmid lineage responsible for the dissemination of the *rmtB* and/or *bla*_CTX–MS_ genes in *Enterobacteriaceae* (Deng et al., 2011; Hou et al., 2012). However, reports of *tet*(M)-harboring IncF plasmids are very scarce. Interesting, pTS14, pST90-1, and pYSP8-1-CTX-M-14 are the only three IncF2 plasmids known to carry the *tet*(M) gene (Figure 1). Several other plasmids harboring the *tet*(M) gene have been reported, such as the IncHI2-type plasmids p1106 (MG825373) and pECAZ147_2 (CP018993), pTW4-IncHI2 (MK293945), and the hybrids IncN1-IncHI2 pHN6DS (MH459020), and IncX1-FI:A:B pYPE12 (CP041443). In addition, pTS14 also exhibited high homology to other IncF2-type plasmids: i.e., the *bla*_CTX–M–14_-bearing *E. coli* plasmid pCA08 (CP009233) with 99.76% identity at 75% coverage, the *bla*_CTX–M–27_- and the *rmtB*-bearing *Salmonella* plasmid pSI108-1 (CP050770) with 99.97% identity at 73% coverage, and the *bla*_CTX–M–27_- and *rmtB*-bearing *Salmonella* plasmid pGDD25-16 (MH316135) with 99.97% identity at 71% coverage (Figure 1).

**FIGURE 2:**
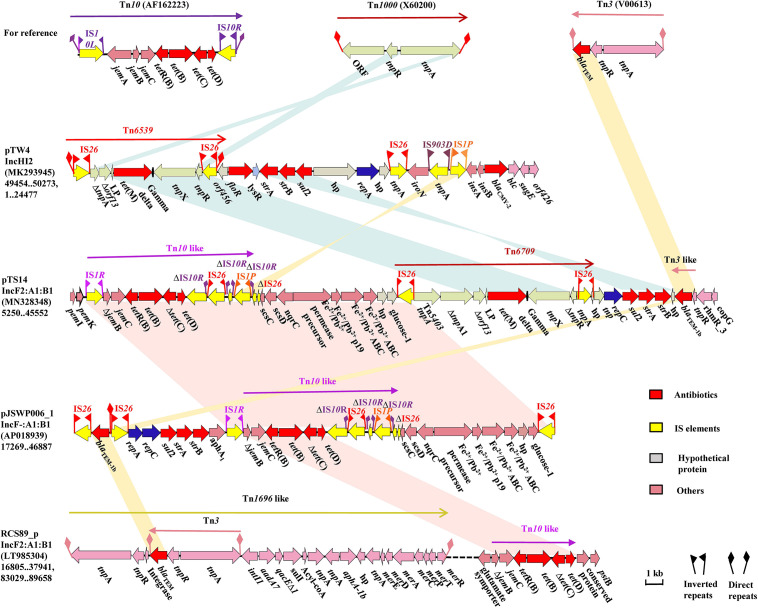
The MDR region of plasmid pTS14 and comparisons with related regions. Genes, mobile elements and other features are colored based on functional classifications. Numbers in parentheses show the nucleotide positions within the corresponding plasmids. Shaded regions denote homologous DNA regions (>97% nucleotide identity). Resistance genes are in red; mobile elements are in yellow; hypothetical proteins are in gray, and others in pink.

Comparative analysis revealed that the most noticeable difference among these IncF2 plasmids lay in the region encoding multidrug resistance (MDR) and virulence factors. The MDR region of pTS14 is composed of several segments, successively including a truncated transposon Tn*10*-like segment carrying the *tet*(B) and *tet*(D) genes, a Feo system for ferrous iron utilization, a *tet*(M)-containing segment, a resistance cluster of *sul*2-*strA*-*strB*, and an incomplete Tn*3*-harboring *bla*_TEM–1b_ transposon (Figure 2). The truncated Tn*10*-like transposon, the first segment, has undergone deletion of IS*10L* and *jem*A, which are located upstream of the truncated *jemB*, and another IS*10R* that was divided into three segments, which were disrupted by the insertion of IS*26* and IS*1P* with 8-bp direct repeats. The similar structure was observed in plasmid pJSWP006-1 (AP018939), and differed by deleting the IS*10R* in RCS89-p (LT985304) (Figure 2). This segment along with the Feo system exhibited high (99.99%) identity with those of plasmid pJSWP006-1 (IncF-:A1:B1, AP018939) with the exception of a three-nucleotide substitution (Figure 2). The *sul*2-*strA*-*strB* module, followed by an incomplete Tn*3* transposon carrying *bla*_TEM–1b_, as the last segment, was located downstream of the *tet*(M)-containing segment. However, an incomplete Tn*3* transposon carrying *bla*_TEM–1b_ and *rmtB* was located upstream of the *qepA1* in plasmid pST90-1 (Supplementary Figure S2).

**FIGURE 3:**
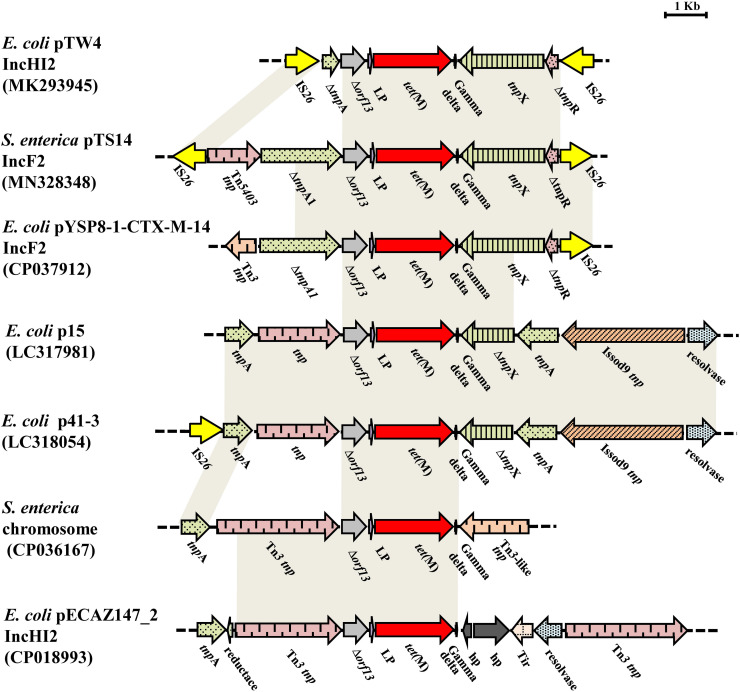
Comparisons of the genetic environment of Tn*6709* harboring *tet*(M) in MN328348, with other sequences carrying *tet*(M) retrieved from the GenBank database. Similar regions are indicated by dotted lines (hp, gene encoding hypothetical protein; lp, gene encoding *tet*(M) leader peptide).

The *tet*(M)-bearing segment, an IS*26*-bracked composite transposon, was designated as Tn*6709* using the Transposon Nomenclature Database^5^. Tn*6709* consists of the insertion sequence IS*26*, an incomplete transposase *tnpA* from Tn*5403*, the incomplete transposase *ΔtnpA* from the Tn*3* family, truncated *orf13*, the *tet*(M) leader peptide (LP) gene, the tetracycline resistance gene *tet*(M), the *tnpX* and *ΔtnpR* genes from the Tn*1000* transposon, and the IS*26* element (Figure 3). Comparative sequence analysis showed that fragment *Δorf13*-*lp*-*tet*(M) was relatively conserved with 100% sequence identity to the other six sequences displayed in Figure 3. Interestingly, the 3′-terminal end of the partial or complete sequence encoding a Tn*3*-family transposase was located upstream of Δ*orf13* in six similar sequences and Tn*6709*, indicating that the transfer of *tet*(M) was closely related to that of the Tn*3* family. Besides, for Tn*6709*, only two sequences (p41-3 and pTW4) carried the IS*26* element upstream of the *tnpA* or *tnpA1* gene. Furthermore, the IS*26* element was located downstream of the incomplete *tnpR* in plasmids pTW4, pYSP8-1-CTX-M-14, and pTS14. The IS*26* element plays a key role in the reorganization of the MDR region of plasmids (Harmer et al., 2014). In this study, reverse PCR was performed to explore the existence of a circular intermediate of the IS*26*-bracked composite transposon Tn*6709*. However, no circular intermediate could be obtained from strain S14. In addition, sequence analysis revealed that there were no direct repeats flanking the IS*26* element in transposon Tn*6709*, indicating that the IS*26*-bracked composite transposon Tn*6709* acquired by pTS14 may have occurred by recombination rather than transposition.

The *tet*(M) gene was originally designated by Burdett et al. (1982), and was subsequently found to be frequently connected with transposons in Gram-positive bacteria, such as the Tn*916* transposon of *Streptococcus faecalis* (Roberts and Kenny, 1987), the Tn*5801*-like transposons of *Staphylococcus aureus* and *Enterococcus faecalis* (de Vries et al., 2009), and the Tn*5397*-like transposons of *Clostridioides difficile* and *Enterococcus faecium* (Agerso et al., 2006). In Gram-negative bacteria, the *tet*(M) gene was first reported in *E. coli* in 2006 as being flanked by IS*26* and IS*Vs1* (Jones et al., 2006), and was first described in *S. enterica* isolated from chicken and pig feces in China in 2017 (Ma et al., 2017). The *tet*(M) gene was found to be associated with the Tn*6539* transposon (Sun et al., 2018). In the present study, the *tet*(M) gene was located on the novel composite transposon Tn*6709* in IncF2:A1:B1, related to the epidemic plasmid lineage IncF2. Taken together, these results suggest that the *tet*(M) gene can transfer from Gram-positive bacteria to Gram-negative bacteria and disseminate between different plasmid groups.

Comparative analysis demonstrated that the *tet*(M)-bearing plasmid pTS14 harbored a Feo system responsible for ferrous iron transportation (Figure 2), while the *tet*(M)-bearing plasmids pST90-1 and pYSP8-1-CTX-M-14 were absent. Interesting, plasmids pST90-1 and pYSP8-1-CTX-M-14 both contained aerobactin (*iut*A-*iuc*ABCD) and Sit (*sit*ABCD) loci (Figure 1 and Supplementary Figure S2). Iron acquisition systems play important roles in colonization and pathogenicity of many bacteria, particularly the aerobactin (*iut*A-*iuc*ABCD), Sit (*sit*ABCD) and Feo system loci (Sabri et al., 2008; Lau et al., 2016; Khajanchi et al., 2019). In *Salmonella*, the iron acquisition system can be encoded by chromosomal pathogenicity islands (Janakiraman and Slauch, 2000) or plasmid genes (Khajanchi et al., 2017). In *S*. *enterica*, the Sit (*sitABCD*) and aerobactin iron acquisition (*iucABCD-iutA*) systems encoded by the IncFIB plasmid have been well characterized, and are reported to enhance virulence potential of intestinal epithelial cells (Khajanchi et al., 2017). In the present study, the Feo system was encoded by the IncF2:A1:B1 plasmid, which may be associated with other biological traits. Thus, the stability of the *tet*(M)-harboring plasmid was further assessed in *E. coli* strain TS14.

### The Biological Characteristics of the Plasmid pTS14

**FIGURE 4:**
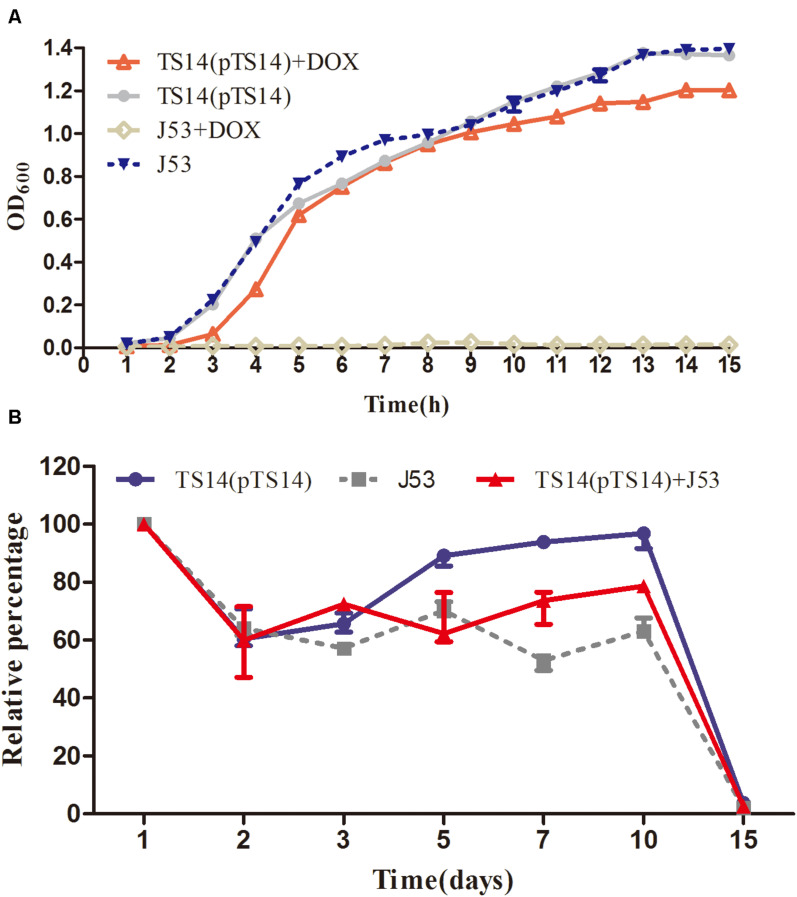
Growth kinetics of the transconjugant TS14 and recipient *E. coli* J53. **(A)** The growth kinetics of transconjugant TS14 harboring pTS14 and recipient *E. coli* J53 over a 15-h inoculation period in the presence and absence of doxycycline (16 μg/mL). **(B)** The starvation survival of J53, TS14, and their mixture. The values are presented as the mean ± standard deviation.

In the bacterial growth experiment, the growth rate of the transconjugant TS14 was similar to that of the recipient *E. coli* J53 in LB broth without doxycycline, while that with doxycycline was slightly lower than the recipient in LB broth without doxycycline (Figure 4A). The plasmid pTS14 existed stably in transconjugant TS14 for at least 14 days of passage in an antibiotic-free environment. The survival rate of TS14 was highest, followed by the mixture of TS14 and *E. coli* J53, and lowest for *E. coli* J53, which demonstrates that the growth of the transconjugant TS14 harboring pTS14 was not lower than that of *E. coli* J53. After 15 days, the bacterial numbers of the three cultures all decreased to less than 10% (Figure 4B). Overall, in this initial study, plasmid pTS14 carrying the Feo system did not affect bacterial growth, although there was no benefit to the transconjugant harboring pTS14. Further investigations are warranted to elucidate the role of the Feo acquisition system of plasmids in *Salmonella*.

## Conclusion

In summary, the self-transmissible IncF2:A1:B1 plasmid pTS14 from *Salmonella* carried the *tet*(M), *tet*(B) and *tet*(D) genes, along with the *sul*2-*strA*-*strB* module and the incomplete *bla*_TEM–1b_-bearing transposon Tn*3*. The *tet*(M) gene was located on the novel composite transposon Tn*6709* and flanked by two IS*26* elements oriented in the opposite direction. In addition, the plasmid pTS14 harbored a Feo system responsible for ferrous iron transportation.

The epidemic IncF2 plasmids have the potential to carry both virulence and antimicrobial resistance determinants, which might contribute to the further dissemination of the *tet*(M) gene via co-selection by other antimicrobials. Therefore, there is a need to monitor the dissemination of this MDR- and virulence-associated plasmid among *Enterobacteriaceae*.

## Data Availability Statement

The raw data supporting the conclusions of this article will be made available by the authors, without undue reservation.

## Author Contributions

GH and YP conceived and designed the experiments. YL, XL, and XC performed the experiments. MC, JL, and YL analyzed the data. SL, DH, and LY contributed to reagents, materials, and analysis tools. YL and GH wrote the manuscript. All authors contributed to the article and approved the submitted version.

## Conflict of Interest

The authors declare that the research was conducted in the absence of any commercial or financial relationships that could be construed as a potential conflict of interest.

## Footnotes

1 http://faculty.washington.edu/marilynr/

2 http://mlst.warwick.ac.uk

3 http://blast.ncbi.nlm.nih.gov/blast

4 https://cge.cbs.dtu.dk/services/PlasmidFinder/

5 https://transposon.lstmed.ac.uk/

## Supplementary Material

FIGURE S1 S1-PFGE **(A)** and Southern hybridization **(B)** of *Salmonella* strain S14 and the corresponding transconjugant TS14 with the *tet*(M) gene as a probe. Marker, Salmonella Braenderup H9812.

FIGURE S2 Comparative analysis of pTS14 with other IncF-type plasmids. Homologous segments generated by a BLASTn comparison (≥97% identity) are shown as gray boxes. Genes are represented by thick arrows. The color code equates to that described in this figure legend.

TABLE S1 The MICs of S14 and transconjugant in this study.
