# Supplementary figures and images for: Characterization of pTS14, an IncF2:A1:B1 Plasmid Carrying tet(M) in a Salmonella enterica Isolate
Source: Front Microbiol. 2020 Jul 3;11:1523. doi: 10.3389/fmicb.2020.01523 (PMC7347964; doi:10.3389/fmicb.2020.01523)

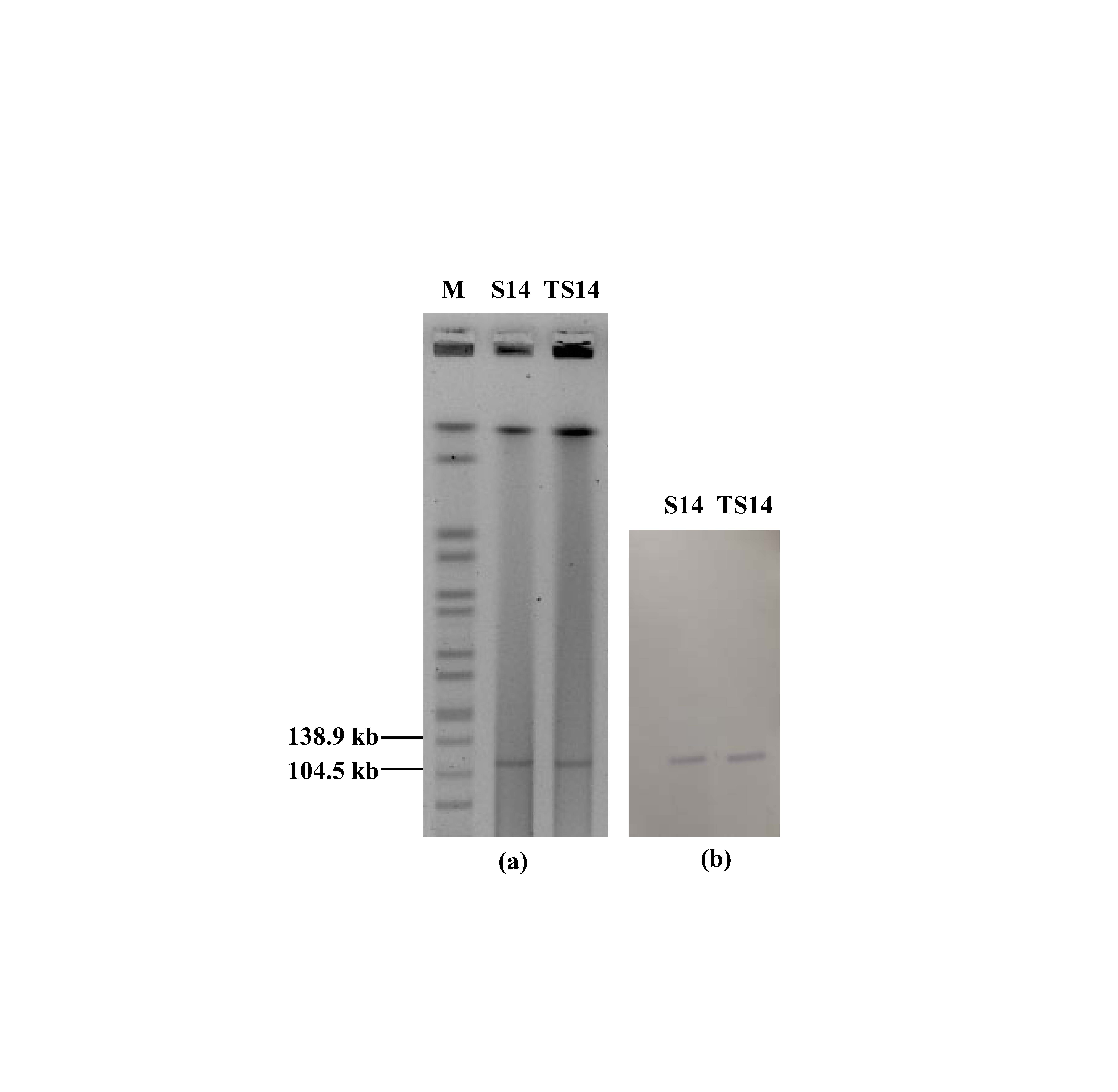

Supplement: FIGURE S1 — S1-PFGE (A) and Southern hybridization (B) of Salmonella strain S14 and the corresponding transconjugant TS14 with the tet(M) gene as a probe. Marker, Salmonella Braenderup H9812. [file Image_1.JPEG]

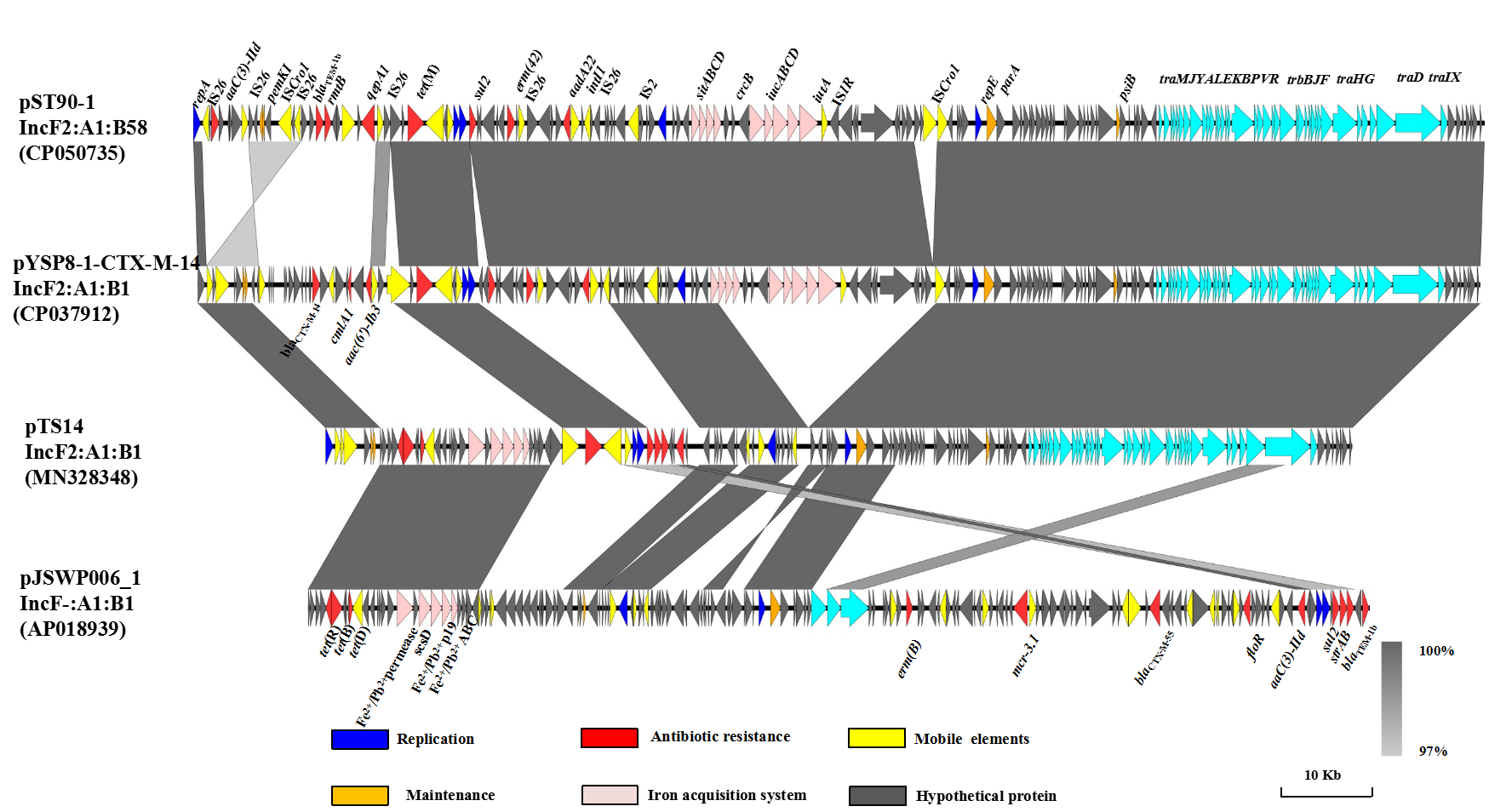

Supplement: FIGURE S2 — Comparative analysis of pTS14 with other IncF-type plasmids. Homologous segments generated by a BLASTn comparison (≥97% identity) are shown as gray boxes. Genes are represented by thick arrows. The color code equates to that described in this figure legend. [file Image_2.TIF]
