# Supplementary material for: Characterization of pTS14, an IncF2:A1:B1 Plasmid Carrying tet(M) in a Salmonella enterica Isolate
Source: Front Microbiol. 2020 Jul 3;11:1523. doi: 10.3389/fmicb.2020.01523 (PMC7347964; doi:10.3389/fmicb.2020.01523)
Supplement: TABLE S1 — The MICs of S14 and transconjugant in this study. [file Table_1.DOCX]

Table S1. The MICs of S14 and transconjugant in this study

| Isolates | AMC | CF | CEQ | GM | AMK | TET | OXY | DOX | FFC | COL | ST | ENR |
| --- | --- | --- | --- | --- | --- | --- | --- | --- | --- | --- | --- | --- |
| S14 | >512 | 4 | 1 | 16 | 2 | 64 | 512 | 128 | >512 | <0.5 | 512 | 1 |
| TS14 | >512 | <0.5 | <0.5 | <0.5 | 1 | 16 | 64 | 64 | 4 | <0.5 | 64 | <0.5 |
| J53 | 1 | <0.5 | <0.5 | <0.5 | 1 | 1 | 1 | 0.5 | 1 | <0.5 | 1 | <0.5 |
| TS14-JS | >512 | <0.5 | 0.5 | 1 | <0.5 | 16 | 64 | 64 | 4 | <0.5 | 64 | <0.5 |
| JS-500 | 1 | <0.5 | 0.5 | 1 | <0.5 | 1 | 1 | 0.5 | 1 | <0.5 | 1 | 0.5 |

AMC, Amoxicillin; CF ceftiofur; CEQ, Cefquinome; GM, Gentamicin; AMK, amikacin; TET, Tetracycline; OXY, oxytetracycline; DOX, doxycycline; FFC, florfenicol; COL, Colistin; ST, trimethoprim/sulfamethoxazole; ENR, Enrofloxacin.
